# Neutrophil extracellular traps in antifungal immunity: mechanisms, dual roles, regulatory networks, and clinical perspectives

**DOI:** 10.3389/fcimb.2026.1908311

**Published:** 2026-09-14

**Authors:** Han Li, Guanzhi Chen, Lingyue Sun

**Affiliations:** Department of Dermatology, The Affiliated Hospital of Qingdao University, Qingdao, China

**Keywords:** antifungal immunity, immune evasion, invasive fungal diseases, NETosis, neutrophil extracellular traps

## Abstract

Invasive fungal diseases (IFDs) are associated with substantial morbidity and mortality. Neutrophil extracellular traps (NETs) are a key effector mechanism of innate antifungal immunity, but they may also act as a double-edged sword. This review summarizes the biological basis of NETs, with an emphasis on lytic and non-lytic NET formation. We discuss how fungal species, morphotypes, cell wall components, and virulence factors regulate NET formation through pattern-recognition receptors (PRRs) and downstream signaling pathways. We also summarize the mechanisms by which pathogenic fungi evade NET-mediated host defense, including immune masking, virulence modulation, signaling interference, and competition for essential nutrients. Furthermore, we discuss how features of the tissue microenvironment, such as hypoxia, hemorrhage, co-infection, and metabolic status, modulate the protective and pathological functions of NETs. We then examine the diagnostic potential of NET-associated biomarkers and highlight therapeutic approaches aimed at modulating NET formation and clearance. Finally, we discuss unresolved issues related to cellular heterogeneity, molecular regulatory networks, and clinical translation, and provide perspectives on how multi-omics approaches, single-cell technologies, and precision immunomodulation may inform future research of IFDs. Overall, this review provides a conceptual framework for exploring NET-targeted diagnostic and therapeutic strategies against IFDs.

## 1 Introduction

Invasive fungal diseases (IFDs) represent a major global public health challenge, with an estimated 6.5 million invasive fungal infections and approximately 3.8 million associated deaths occurring worldwide each year (Denning, 2024). In 2022, the World Health Organization (WHO) released its first fungal priority pathogens list, in which *Aspergillus fumigatus, Candida albicans, Candida auris*, and *Cryptococcus neoformans* were classified in the critical priority group (World Health Organization, 2022). IFDs predominantly affect immunocompromised populations, including patients with hematological malignancies, solid organ transplant recipients, and critically ill individuals receiving immunosuppressive therapy. In recent decades, the epidemiology of IFDs is also evolving with the emergence of antifungal-resistant pathogens and the expanding population of immunocompromised patients receiving novel therapies (Giannella et al., 2025). However, the clinical management of IFDs currently faces two major challenges. First, in terms of diagnosis, many existing diagnostic approaches are limited by suboptimal sensitivity, limited accessibility outside specialized laboratories, and delayed turnaround times, highlighting the need for rapid, accessible, and accurate diagnostic tools (Murtagh et al., 2026). Second, in terms of treatment, conventional antifungal therapy is increasingly challenged by antifungal resistance (Cornely et al., 2025).

At the host-defense level, fungal cell wall components are recognized by pattern-recognition receptors (PRRs), which initiate downstream signaling and coordinate antifungal immune responses (Carrillo-Serradell et al., 2025). Neutrophils contribute to antifungal immunity partly through the formation of neutrophil extracellular traps (NETs) (Brinkmann et al., 2004; Urban et al., 2006). However, excessive or dysregulated NET formation may exacerbate inflammatory tissue injury, reflecting the context-dependent double-edged nature of NET responses (Brinkmann and Zychlinsky, 2012; Mutua and Gershwin, 2021).

Although increasing evidence supports important roles for NETs in fungal infections, their functional outcomes across different infectious contexts remain incompletely understood (Urban and Nett, 2019). Differences in fungal species and morphotype may influence the magnitude and functional consequences of NET responses (Branzk et al., 2014; Urban and Nett, 2019). Differences in the methods used to detect and quantify NET formation, including extracellular DNA measurements, NET-associated molecular markers, and microscopy-based assessment, may further contribute to variation in the magnitude of NET responses reported across studies (Stoimenou et al., 2022). However, the factors that determine the balance between protective NET-mediated antifungal activity and NET-associated immunopathological damage remain incompletely defined (Urban and Nett, 2019). Importantly, some canonical mechanisms of NET formation were initially defined using non-fungal stimuli or other infectious and inflammatory models (Fuchs et al., 2007; Yousefi et al., 2009; Pilsczek et al., 2010), and their relevance may vary across fungal species and experimental settings (He et al., 2022).

Rather than providing another general overview of NETosis biology or canonical signaling pathways, this review focuses on the context-dependent determinants that shape NET functional outcomes during fungal infection. We integrate fungal species and morphotype, recognition and signaling pathways, fungal immune-evasion strategies, host immune status, and local microenvironmental factors within a unified framework to explain why NET responses may contribute to either antifungal protection or immunopathological tissue injury. We also critically examine discrepancies among experimental studies, including differences in fungal models, neutrophil sources, NET-detection methods, and antifungal readouts, and distinguish mechanisms directly demonstrated in fungal infection models from those extrapolated from other inflammatory settings. Finally, we discuss the diagnostic and therapeutic implications of this functional heterogeneity, with particular emphasis on the challenges of selectively targeting pathological NET responses while preserving protective antifungal immunity.

## 2 Biological basis of NETs

In addition to classical antimicrobial mechanisms such as phagocytosis, degranulation, and the production of reactive oxygen species (ROS), neutrophils contribute to host defense through the release of NETs. NETs are web-like extracellular structures composed of a decondensed chromatin scaffold associated with antimicrobial proteins, including neutrophil elastase (NE), myeloperoxidase (MPO), and histones. NETs exert antimicrobial effects partly by capturing and immobilizing pathogens, concentrating antimicrobial molecules at sites of infection, and inactivating pathogen virulence factors, thereby limiting pathogen dissemination and modulating local inflammatory responses (Brinkmann et al., 2004). Subsequent studies have revealed that NETs are also involved in antifungal immunity, with evidence from several fungal infection models, including *Candida albicans* and *Aspergillus fumigatus*, suggesting their potential roles in limiting fungal growth and dissemination (Urban et al., 2006; McCormick et al., 2010).

Based on the mode of NET release and the fate of neutrophils, NET formation has commonly been described as involving lytic and non-lytic modes. Classical suicidal NETosis represents a lytic pathway (Fuchs et al., 2007), whereas rapid NET release without immediate neutrophil lysis has also been described (Pilsczek et al., 2010). Furthermore, mitochondrial DNA-derived NETs (mtNETs) have been reported, suggesting additional heterogeneity in NET-release mechanisms (Yousefi et al., 2009).

During classical suicidal NETosis, neutrophils undergo a series of morphological changes following stimulation. Chromatin first decondenses, followed by the disruption of nuclear and granule membranes and the mixing of nuclear and granular components, allowing antimicrobial proteins to become incorporated into DNA-containing NET structures. Plasma membrane rupture subsequently occurs, releasing these complexes into the extracellular space, where they form web-like NET structures. This process depends on nicotinamide adenine dinucleotide phosphate (NADPH) oxidase (NOX)-mediated ROS production and represents a distinct cell-death process that differs from apoptosis and necrosis (Fuchs et al., 2007).

In contrast to classical lytic NETosis, rapid non-lytic NET release has been described in response to *Staphylococcus aureus*. In this model, nuclear DNA is packaged into vesicles and released extracellularly without immediate neutrophil lysis or plasma membrane rupture (Pilsczek et al., 2010). These findings differ from the slower, ROS-dependent lytic process described by Fuchs et al., indicating that NET-release kinetics and cellular fate can vary substantially according to the inducing stimulus (Fuchs et al., 2007; Pilsczek et al., 2010). Mitochondrial DNA-containing NETs (mtNETs) can also be released by viable neutrophils. In the model described by Yousefi et al., GM-CSF priming followed by short-term C5a receptor or TLR4 stimulation induced ROS-dependent release of NETs containing mitochondrial, but not nuclear, DNA without requiring neutrophil death (Yousefi et al., 2009).

Together, these studies indicate that NET release is not a uniform process but varies according to the inducing stimulus, release kinetics, DNA source, ROS dependence, and neutrophil fate (Fuchs et al., 2007; Yousefi et al., 2009; Pilsczek et al., 2010). Because these mechanistic distinctions were largely established using non-fungal stimuli, their applicability to fungal infection should be interpreted in a context-dependent manner (He et al., 2022).

Differences in fungal species and experimental stimulation conditions may further contribute to variation in reported NET responses (Urban and Nett, 2019; He et al., 2022).

## 3 Mechanisms of NET induction

Neutrophils can tailor their antimicrobial responses to microbial size through a mechanism involving Dectin-1-mediated phagocytosis (Branzk et al., 2014). In the model described by Branzk et al., large, non-phagocytosable microbes, including *C. albicans* hyphae, preferentially induced NET release, whereas phagocytosis of smaller microbes suppressed NET formation by limiting the nuclear translocation of neutrophil elastase (NE) (Branzk et al., 2014). Large *C. albicans* hyphae can also induce neutrophil swarming, a coordinated multicellular response characterized by calcium signaling and the accumulation of neutrophils around fungal targets (Phillips et al., 2025). SYK, BTK, CD18, and MPO contribute to effective swarming, which restricts hyphal growth (Phillips et al., 2025).

### 3.1 Regulation of NET formation by fungal species, components and morphotypes

The ability to induce NET formation differs markedly among fungal species. *C. albicans (*Urban et al., 2006), *A. fumigatus (*McCormick et al., 2010), *Histoplasma capsulatum (*Thompson-Souza et al., 2020), *Phialophora verrucosa (*Liu et al., 2021), and *Paracoccidioides brasiliensis (*Mejía et al., 2015) have all been shown to induce NET formation. In contrast, *Alternaria alternata* induced only limited NET formation in the experimental setting examined by Shin et al (Shin et al., 2023). Notably, NET responses can also differ within the same fungal species. In *C. neoformans*, the acapsular CAP67 strain induced NET formation, whereas the encapsulated wild-type B3501 strain did not; notably, the capsular polysaccharides showed divergent effects, with GXMGal inducing NET release and GXM suppressing NET formation (Rocha et al., 2015). These findings indicate that NET-inducing capacity can vary not only among fungal species but also according to pathogen-specific structural features.

Fungal structural components, secreted products, and physical characteristics can differentially regulate NET formation.

Different *C. albicans* components can induce NET formation with varying efficiencies. Purified cell-wall mannans and β-glucans, as well as secreted aspartic proteases (Saps), induce NET release, with the hypha-associated Sap4 and Sap6 showing the strongest NET-inducing activity among the Saps tested (Zawrotniak et al., 2017). Extracellular nucleic acids provide another NET-inducing signal. Biofilm-associated extracellular nucleic acids and purified *C. albicans* DNA and RNA induce NET release by human neutrophils, with NET formation induced by purified fungal nucleic acids depending on endocytosis and NADPH oxidase-mediated ROS production (Smolarz et al., 2021). TLR8 contributes to RNA-induced NETosis, whereas the receptor responsible for fungal DNA recognition remains unresolved (Smolarz et al., 2021). Candidalysin further illustrates that extracellular chromatin responses to an isolated fungal factor may differ from those elicited by the intact pathogen. Candidalysin-producing *C. albicans* hyphae strongly promote canonical NET formation, whereas candidalysin alone induces more compact and less fibrous NET-like structures (NLS) (Unger et al., 2023). NLS formation involves NADPH oxidase-mediated ROS production and Ca²^+^ influx, with Ca²^+^-dependent PAD4 activation promoting histone hypercitrullination; however, candidalysin alone does not trigger lamin A/C phosphorylation required for canonical NET release (Unger et al., 2023). During exposure to candidalysin-producing hyphae, canonical NETs and NLS occur concurrently and form extracellular networks that entangle fungal hyphae and inhibit their growth (Unger et al., 2023). Thus, NLS induced by candidalysin alone should not be equated with canonical NET responses elicited by intact fungal cells.

The influence of fungal morphotype on NET induction may also vary with experimental conditions. In *C. albicans*, Branzk et al. found that large hyphae induced NET release, whereas a yeast-locked *hgc1Δ* mutant failed to induce NETs in human peripheral blood neutrophils (Branzk et al., 2014). In contrast, Kenno et al. observed rapid NET release in response to both yeast and hyphal cells, although hyphae induced larger amounts of NETs and maintained this capacity for up to 4 h, whereas the NET-inducing capacity of yeast cells was lost at 4 h (Kenno et al., 2016). Ermert et al. similarly found that *C. albicans* hyphae elicited stronger NET responses than yeast cells in mouse neutrophils (Ermert et al., 2009). Collectively, these studies consistently identify hyphae as stronger NET stimuli, whereas the NET-inducing capacity of yeast appears more dependent on the experimental model and conditions.

Morphotype-dependent differences also vary across fungal species. In *A. fumigatus*, NET formation is morphotype dependent, with hyphae inducing more pronounced NET responses than conidia (McCormick et al., 2010). In *Sporothrix*, by contrast, both conidia and yeast-like cells induce NET formation, but yeast-like cells are stronger NET stimuli (Galván-Hernández et al., 2023). Thus, morphotype-dependent NET induction does not follow a uniform pattern across fungi and should be interpreted in relation to both species-specific biology and experimental design.

### 3.2 Regulation by pattern-recognition receptors and signaling pathways

Neutrophils recognize fungal-associated molecular patterns (PAMPs) through multiple pattern-recognition receptors (PRRs) expressed on their surface and initiate distinct signaling responses depending on fungal cell wall composition and morphological characteristics. Different receptors recognize specific fungal components and activate downstream signaling pathways, thereby regulating NET formation. Among these receptors, C-type lectin receptors (CLRs) represent a major class of PRRs involved in fungal cell wall recognition. Dectin-1 and Dectin-2 are well-characterized CLR members involved in antifungal immunity and NET regulation and are discussed here as representative examples.

Dectin-1 is a major pattern-recognition receptor for fungal β-glucans and contributes to β-glucan-induced NET formation. In human peripheral blood neutrophils, Dectin-1 blockade reduced NET formation and attenuated NOX-associated ROS production as well as ERK and p38 MAPK activation, supporting the involvement of Dectin-1-dependent ROS/MAPK signaling in this response (Zhang et al., 2025). Separately, β-glucan particle-induced NET formation has been shown to involve Src-family kinases and Syk (Nanì et al., 2015). Because β-glucans can engage multiple neutrophil receptors, however, the extent to which this Src/Syk-dependent NET response is specifically attributable to Dectin-1 remains unclear.

Fungus-induced NET formation does not appear to follow a universal signaling program, and the relative contribution of individual pathways may vary across fungal and experimental contexts. In human neutrophils stimulated with *H. capsulatum* yeasts, earlier work identified an oxidative NET response involving CD18 and Src/Syk signaling (Thompson-Souza et al., 2020). Subsequent mechanistic analysis further revealed distinct requirements for PI3K isoforms: PI3Kδ was required for both ROS production and NET formation, whereas PI3Kγ contributed to NET formation but was dispensable for ROS generation (Thompson-Souza et al., 2026). Together, these findings indicate that, even within a single fungal model, the signaling requirements for NET formation and ROS production can be partially dissociated.

Dectin-2 recognizes high-mannose structures on fungal cells, including *C. albicans (*McGreal et al., 2006). Because Dectin-2 lacks an intrinsic intracellular signaling motif, it associates with the Fc receptor γ-chain (FcRγ) to transmit activating signals (Sato et al., 2006). In neutrophils challenged with unopsonized *C. albicans*, Dectin-2 mediates NADPH oxidase-independent NET formation through a Syk–Ca²^+^–PKCδ signaling pathway, with PAD4 activation and NE nuclear translocation contributing to chromatin decondensation and NET release (Wu et al., 2019). These findings demonstrate that fungal recognition can engage mechanistically distinct NET pathways depending on the receptor and stimulation context.

CARD9 (caspase recruitment domain-containing protein 9) is a key adaptor downstream of C-type lectin receptor signaling and contributes to antifungal immunity (Gross et al., 2006). In a *C. albicans* infection model, Loh et al. showed that loss of the negative regulator Dok3 enhanced CARD9 signaling and was accompanied by increased neutrophil NETosis and other antifungal effector functions (Loh et al., 2019). However, because this study manipulated Dok3 rather than CARD9 itself, the observed increase in NETosis does not establish a direct role for CARD9 in the execution of NET formation. In murine mucormycosis models, Card9 deficiency was associated with increased susceptibility to *Rhizopus arrhizus* infection (Sun et al., 2019), while subsequent work showed that Card9-deficient neutrophils released fewer NETs in response to *Mucor irregularis* hyphae (Sun et al., 2021). Thus, current evidence supports an association between CARD9-regulated antifungal signaling and NET responses.

Beyond CLR-mediated recognition, other receptors and inflammatory pathways can also shape fungus-induced NET responses. For example, CR3 (CD11b/CD18) was required for β-glucan- and *A. fumigatus*-induced NET formation in the model examined by Clark et al (Clark et al., 2018). Fungal recognition can also activate Syk-coupled NLRP3 inflammasome signaling and IL-1β production (Gross et al., 2009), although its direct contribution to NET formation during fungal infection remains less well defined. Outside fungal infection models, cytokines such as TNF-α have also been shown to promote NET formation under inflammatory conditions (Keshari et al., 2012), but the extent to which such cytokine-mediated regulation shapes antifungal NET responses requires further investigation.

**Figure 1:**
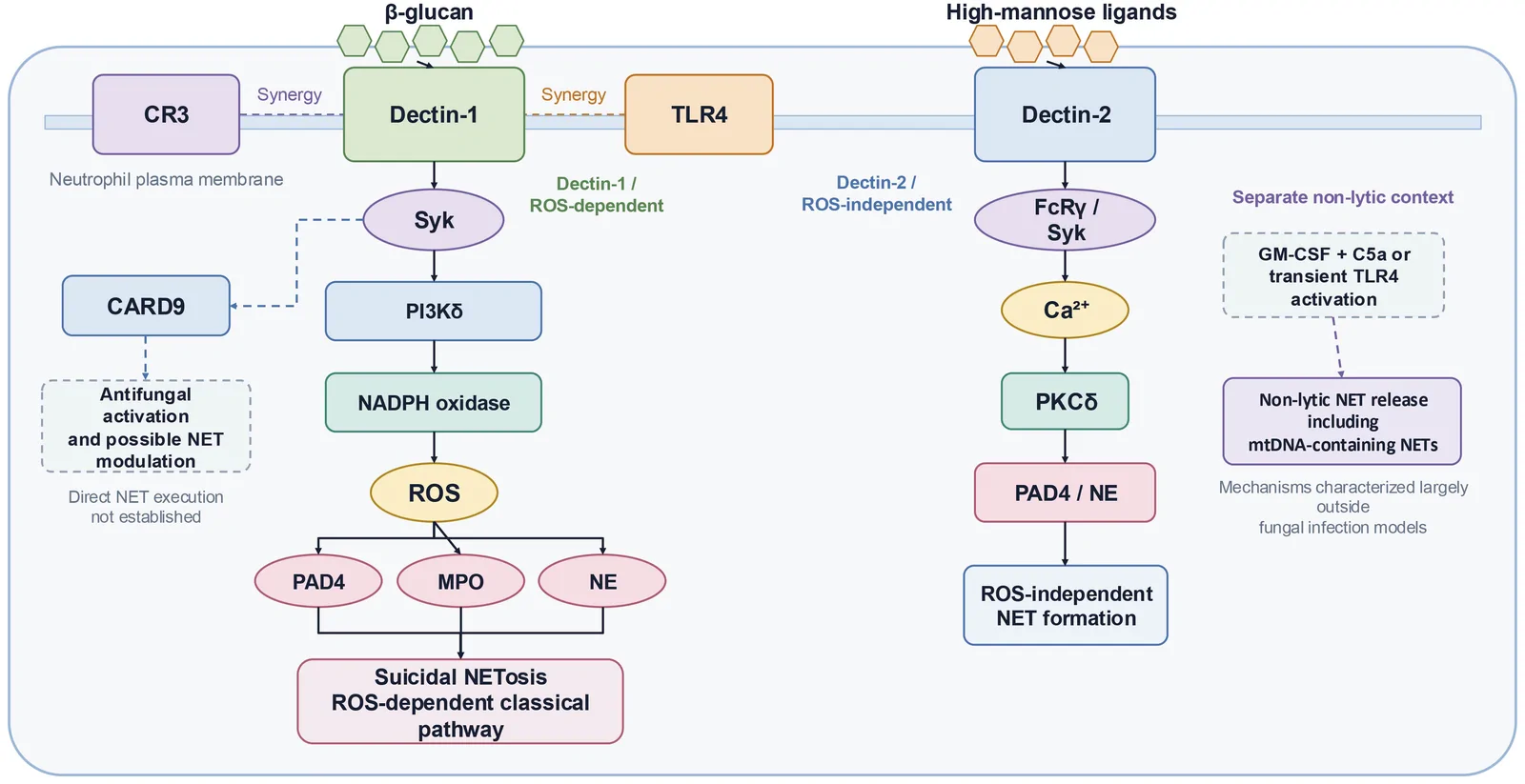
Fungal recognition and signaling pathways regulating neutrophil extracellular trap (NET) formation. Solid arrows indicate established signaling relationships, whereas dashed arrows represent synergistic interactions or context-dependent regulatory mechanisms.

Collectively, current evidence indicates that fungus-induced NET formation is shaped by fungal morphotype, surface composition, receptor engagement, and the surrounding inflammatory context rather than by a single conserved signaling pathway. However, direct mechanistic evidence remains uneven across fungal species, and some NET-regulatory mechanisms are still inferred from non-fungal experimental systems. The major fungal recognition receptors and downstream signaling pathways implicated in NET formation are summarized in **Figure 1**.

## 4 Protective functions of NETs

NETs can contribute to antifungal defense by trapping fungal cells and concentrating antimicrobial components at sites of infection. In an early study, *C. albicans* induced NET formation, and the resulting NETs captured and killed both yeast and hyphal forms through NET-associated neutrophil granule components (Urban et al., 2006). However, the protective effects of NETs do not necessarily depend on direct fungal killing. In a blood-based microfluidic system, NETs captured *C. albicans* yeast cells and delayed their transition to the hyphal form (Sakuma et al., 2025).

During *Aspergillus fumigatus* infection, NETs can restrict germ-tube and hyphal growth (McCormick et al., 2010). Bruns et al. further showed that NET formation varies according to fungal morphotype and is more pronounced in response to hyphae than to conidia (Bruns et al., 2010). However, the antifungal effects of NETs against *A. fumigatus* appear to involve growth restriction more prominently than complete fungal killing (Bruns et al., 2010; McCormick et al., 2010). NETs can also function cooperatively with other neutrophil responses. During neutrophil swarming around *Candida* clusters, MPO and NADPH oxidase contribute to delaying fungal germination, whereas NET release within the swarm helps restrict subsequent fungal growth (Hopke et al., 2020). Together, these findings suggest that NET-mediated antifungal activity can involve direct killing, growth restriction, and coordinated containment, with the relative contribution of these effects varying across fungal and experimental contexts.

Beyond direct antimicrobial effects, NETs may also modulate neutrophil inflammatory functions. In a non-fungal *in vitro* model, exposure of human neutrophils to isolated NETs induced IL-8 and BAFF secretion, suggesting a potential mechanism by which NETs could amplify inflammatory responses and influence innate–adaptive immune crosstalk (Dömer et al., 2021). However, whether this feedback mechanism contributes directly to antifungal protection remains to be established.

Beyond physical trapping and antimicrobial protein-mediated killing, NETs can also contribute to antifungal defense through nutritional restriction. Urban et al. identified calprotectin as an antimicrobial component of NETs and demonstrated that NET-associated calprotectin contributes to antifungal activity against *C. albicans* (Urban et al., 2009).

Importantly, the apparently divergent conclusions regarding NET-mediated fungal killing versus containment may reflect biological and methodological heterogeneity among studies. Fungal species and morphotype, experimental model, and the antifungal endpoint assessed may all influence the apparent functional outcome of NET responses. NET induction does not necessarily translate into direct fungicidal activity; for example, *P. brasiliensis* induced NET formation in human neutrophils without a corresponding reduction in fungal viability *in vitro (*Mejía et al., 2015). Moreover, direct killing, physical capture, delayed morphogenesis, and growth restriction represent biologically distinct antifungal endpoints and should not be considered interchangeable measures of NET activity. This distinction is particularly important when comparing studies that use different experimental models and antifungal readouts (Urban et al., 2006; Bruns et al., 2010; Hopke et al., 2020; Sakuma et al., 2025). Thus, at least some of the apparent inconsistencies among studies may reflect differences in fungal biology and experimental methodology rather than fundamentally contradictory functions of NETs. Cross-study comparisons may be further complicated by differences in neutrophil source or functional state and by the use of different NET-detection approaches, which do not necessarily measure equivalent aspects of NET formation.

## 5 Detrimental effects of NETs

Although NETs can contribute to antifungal defense, excessive or persistent NET responses may also promote inflammatory tissue injury in specific fungal disease contexts.

In chronic granulomatous chromoblastomycosis, NET accumulation occurs within a Th2-dominant inflammatory environment associated with persistent fungal infection and tissue fibrosis (Huang et al., 2024). Abundant NETs were observed within granulomatous lesions, where they were associated with the containment of fungal sclerotic cells (Huang et al., 2024). Persistent NET accumulation in this Th2-skewed inflammatory environment was also associated with collagen deposition, lesion fibrosis, and tissue damage (Huang et al., 2024). These observations illustrate that NET-mediated fungal containment and immunopathological tissue injury can coexist within the same chronic infection context.

Chemotherapy-associated neutropenia is an established risk factor for invasive fungal infections, particularly invasive aspergillosis, because reduced neutrophil availability compromises antifungal host defense (Patterson et al., 2016). However, NET responses in this setting are not uniform. In a longitudinal study of patients undergoing chemotherapy, oral mucositis and clinical candidiasis were associated with reduced salivary NET formation, whereas *Candida albicans* colonization was associated with increased NET levels (de Arruda et al., 2024). These findings indicate that NET responses during chemotherapy vary according to the clinical context and should not be interpreted simply as pathological overactivation.

Fungal allergens may also promote pathological NET accumulation in allergic airway inflammation. In a mouse model of severe steroid-resistant neutrophilic airway disease, exposure to the fungal allergen *Alternaria alternata* induced RAGE-dependent neutrophilic inflammation and NLRP3 inflammasome activation (Killian et al., 2023). NET-associated markers, including citrullinated histone H3 and neutrophil elastase, were also elevated following *A. alternata* exposure and were markedly reduced in RAGE-deficient mice (Killian et al., 2023). The same study identified an atypical Siglec-F^+^ neutrophil population in the airways. These findings link RAGE-dependent inflammatory signaling with NET accumulation in this experimental model, although the direct causal contribution of NETs to disease severity remains to be established (Killian et al., 2023).

*Pneumocystis jirovecii pneumonia (PJP)* primarily affects immunocompromised populations, including individuals with HIV/AIDS, cancer, and solid organ transplant recipients (Sayson et al., 2024*).*

In a murine model of *Pneumocystis* pneumonia, *P. murina* infection was associated with increased NLRP3 inflammasome and NETosis-related signaling, and NETs were detected in infected lung tissue (Sayson et al., 2024). P*. murina* also directly induced NET formation in isolated neutrophils, while isolated NETs reduced fungal viability *in vitro (*Sayson et al., 2024). Thus, this study supports an antifungal function of NETs, although excessive neutrophil activation in the inflamed lung may potentially contribute to tissue injury (Sayson et al., 2024).

In contrast, another murine study identified a detrimental NET response during *Pneumocystis* infection. *Pneumocystis* triggered LTB4-dependent neutrophil swarming and the formation of agglutinative NETs, which failed to eliminate the pathogen and impaired neutrophil-mediated fungal killing (Zhou et al., 2024). Pharmacological blockade of LTB4 signaling reduced neutrophil accumulation and NET release, enhanced *Pneumocystis* clearance, and attenuated lung injury (Zhou et al., 2024).

The apparently divergent findings of these studies further illustrate the context dependence of NET function: isolated NETs exhibited direct anti-*Pneumocystis* activity *in vitro*, whereas swarming-associated agglutinative NETs impaired fungal clearance and promoted lung injury *in vivo (*Sayson et al., 2024; Zhou et al., 2024). In the latter model, pharmacological blockade of LTB4 signaling reduced swarming-associated NET formation, attenuated pulmonary inflammation, and enhanced *Pneumocystis* clearance (Zhou et al., 2024).

Gut fungal dysbiosis has been shown to play an important role in gallstone formation. Fungus-induced NET formation may also contribute to pathology outside classical sites of fungal infection. In a gut–liver axis model, *Rhodotorula mucilaginosa* promoted hepatic neutrophil activation and NET formation, with NETs contributing to gallstone nucleation and growth (Lu et al., 2026).

Taken together, these findings demonstrate that NET functions in fungal-associated diseases are highly context dependent, with protective and pathological outcomes varying according to fungal species, host environment, experimental setting, and the mode of NET response.

## 6 Fungal immune evasion

In response to NET-mediated host defense, pathogenic fungi have evolved diverse evasion strategies, including modulation of NET induction, disruption of NET integrity, and attenuation of NET-associated antimicrobial activity. These mechanisms vary among fungal species and are influenced by pathogen-specific features such as cell wall composition and virulence factors. Representative mechanisms are discussed below.

*Candida albicans* is one of the most extensively studied models of NET-mediated antifungal immunity. Tseng et al. showed that reduced candidalysin expression in intravascular catheter-associated *C. albicans* biofilms was associated with decreased NETosis and increased resistance to neutrophil-mediated biofilm elimination (Tseng et al., 2024). These findings suggest that downregulation of candidalysin can promote fungal persistence by limiting NET induction.

*Candida albicans* and *C. glabrata* exhibit extracellular 3′-nucleotidase/nuclease (3′NT/NU) activity that promotes NET degradation. Pharmacological inhibition of this activity reduced NET disruption and decreased *C. albicans* survival during interactions with neutrophils, supporting extracellular nucleotide metabolism as a NET-evasion mechanism (Afonso et al., 2021).

In addition, the *C. albicans* aspartyl protease Sap6 can be internalized by neutrophils and impair NADPH oxidase-dependent ROS production, thereby suppressing ROS-dependent NETosis (Zawrotniak et al., 2025).

Within the *C. albicans* biofilm microenvironment, extracellular DNA (eDNA) and extracellular RNA (eRNA) can form complexes with the host antimicrobial peptide LL-37. These complexes suppress neutrophil ROS production and NETosis while increasing IL-8 production, thereby shifting the neutrophil response away from local NET-mediated antifungal activity (Juszczak et al., 2024).

NET-associated calprotectin contributes to antifungal defense against *A. fumigatus*. In patients with chronic granulomatous disease (CGD), gene therapy-mediated restoration of neutrophil function restored NET formation and calprotectin-dependent anti-*Aspergillus* activity (Bianchi et al., 2011). Conversely, the cell wall-associated exopolysaccharide galactosaminogalactan (GAG) enhances fungal resistance to NET-mediated damage. Lee et al. showed that increased cell wall-associated GAG enhanced resistance to NADPH oxidase-dependent NET-mediated damage without reducing NET formation itself (Lee et al., 2015). Neutrophils also employ distinct antimicrobial mechanisms against different *A. fumigatus* morphotypes. Gazendam et al. showed that conidial killing primarily involved CD11b/CD18-dependent, nonoxidative intracellular mechanisms, whereas hyphal killing depended on Fcγ receptor-mediated signaling, NADPH oxidase, and MPO (Gazendam et al., 2016). Although *A. fumigatus* induced NET formation in this model, NETs did not contribute measurably to fungal killing (Gazendam et al., 2016). These findings further indicate that NET formation and antifungal killing should not be considered equivalent outcomes in *A. fumigatus* infection.

Beyond resistance to NET-associated antimicrobial activity, *A. fumigatus* can also adapt morphologically to neutrophil attack. A live-cell imaging study showed that interactions with neutrophils stimulated evasive hyphal branching, redirecting fungal growth away from sites of concentrated neutrophil activity (Ellett et al., 2017), although this response has not been shown to be NET specific.

The polysaccharide capsule of *C. neoformans* represents an important mechanism for suppressing NET formation. Rocha et al. showed that encapsulated *C. neoformans* failed to induce substantial NET release, whereas an acapsular mutant induced robust NET formation. The major capsular polysaccharide glucuronoxylomannan (GXM) also inhibited NET formation, supporting capsule-dependent suppression of NETosis as an immune-evasion mechanism (Rocha et al., 2015).

In contrast to capsule-mediated suppression of NET formation, a recent study showed that *C. neoformans*-derived extracellular vesicles (EVs) induced NET formation through PAD4-, NF-κB/p65-, and NADPH oxidase-associated mechanisms (Cai et al., 2026). In an *in vitro* blood–brain barrier model, EV-induced NETs disrupted endothelial tight junction integrity, reduced claudin-5 and occludin expression, and enhanced fungal adhesion and barrier permeability (Cai et al., 2026).

Collectively, these findings illustrate the context-dependent effects of *C. neoformans* on NET responses: capsule-dependent mechanisms can suppress NET-mediated antifungal defense, whereas EV-induced NET formation may contribute to endothelial barrier dysfunction.

The cell wall-associated protein Gp70 contributes to the interaction between *S. schenckii* and host immune cells. High-level silencing of GP70 altered fungal cell-wall composition, increased β-1,3-glucan exposure, and enhanced both phagocytosis and NET formation by human granulocytes (López-Ramírez et al., 2024). Although Dectin-1 contributed to recognition of the *GP70*-silenced strains, its direct involvement in the increased NET response was not established, indicating that the relationship between Gp70-dependent cell-wall remodeling and NET formation requires further clarification.

Beyond modulation of neutrophil recognition, *S. schenckii* exhibits adaptive responses to oxidative stress. Experimental exposure to superoxide- or H_2_O_2_-generating conditions induces antioxidant and cell-wall-associated responses involving superoxide dismutase (SOD), thioredoxin, peroxiredoxin, and other stress-responsive proteins (Ruiz-Baca et al., 2019; Félix-Contreras et al., 2020; Saucedo-Campa et al., 2022). These responses may enhance fungal tolerance to neutrophil-derived oxidative stress; however, their direct contribution to resistance against NET-mediated antifungal activity remains unclear.

In addition to directly interfering with NET formation or integrity, fungi may adapt to NET-associated nutritional restriction. NETs contribute to the killing of *F. pedrosoi* hyphae, and NET-associated calprotectin can impose zinc limitation during antifungal responses (Breda et al., 2020; Santos et al., 2024). Under experimental zinc deprivation, *F. pedrosoi* upregulated high-affinity zinc acquisition systems, including zinc transporters and the zincophore Pra1, together with cell-wall remodeling and antioxidant defense responses (Santos et al., 2024). Although these adaptations have not been directly demonstrated during NET exposure, they may enhance fungal tolerance to NET-associated nutritional immunity.

The virulent *P. brasiliensis* isolate Pb18 induced looser and more dispersed NET structures than the low-virulence isolate Pb265, which induced denser and more compact NETs. Pb18 also exhibited DNase-like activity and increased expression of candidate DNase-associated genes, particularly *PADG_08285*, during interaction with human neutrophils (Zonta et al., 2020). These findings suggest that DNase-like activity may contribute to NET degradation and fungal escape, although the specific fungal nuclease responsible has not been directly established.

Collectively, pathogenic fungi can resist NET-mediated immunity through multiple mechanisms, including suppression of NET induction, degradation of NET structures, resistance to NET-associated antimicrobial effectors, remodeling or masking of immunogenic cell-wall components, and adaptation to nutrient-restricted environments. These findings indicate that the outcome of NET–fungus interactions depends not only on the extent of NET formation but also on fungal species, virulence-associated traits, and the functional integrity of NET-associated antimicrobial mechanisms. Representative interactions between fungal pathogens and NETs are summarized in **Table 1**.

**Table 1:** Summary of fungal characteristics, neutrophil responses, and NET-associated mechanisms in representative pathogenic fungi.

Fungal species	Receptors	Pathway	Susceptibility to NETs	Escape mechanism	Refs
*Candida albicans*	Dectin-1; TLR2/TLR4; CR3	Syk-associated signaling; NADPH oxidase–ROS and PAD4-dependent NET formation	++	Biofilm extracellular matrix and hypoxia impair NET formation; eDNA/eRNA -LL-37 complexes suppress ROS production and NETosis; 3′-nucleotidase/nuclease (3′-NT/NU) activity promotes NET degradation; reduced candidalysin expression limits NET induction; and Sap6 suppresses NADPH oxidase-dependent ROS production and NETosis	(Urban et al., 2006; Wu et al., 2019; Afonso et al., 2021; Tseng et al., 2024; Juszczak et al., 2024; Juszczak et al., 2025; Zawrotniak et al., 2025)
*Aspergillus fumigatus*	CR3/CD18; Dectin-1-associated recognition	CR3/CD18–Src/Syk–NADPH oxidase–ROS signaling; NET induction and antifungal effects differ between conidia and hyphae	Morphotype-dependent; hyphae induce stronger NET responses (+)	Cell wall-associated galactosaminogalactan (GAG) increases resistance to NET-mediated damage without necessarily reducing NET formation; fungal antioxidant defenses also limit oxidative damage	(Bruns et al., 2010; McCormick et al., 2010; Bianchi et al., 2011; Lee et al., 2015; Gazendam et al., 2016; Clark et al., 2018; Thompson-Souza et al., 2020)
*Cryptococcus neoformans*	Not fully defined	Capsule-dependent modulation: GXMGal and an acapsular strain induce NET release, whereas GXM suppresses it; extracellular vesicles induce NETs through mechanisms associated with PAD4, NF-κB/p65, and NADPH oxidase	Strain-, capsule-, and fungal-component-dependent	The polysaccharide capsule, particularly GXM, suppresses NET induction; fungal-derived extracellular vesicles can instead enhance NET formation and may promote pathological endothelial-barrier injury	(Rocha et al., 2015; Cai et al., 2026)
*Sporothrix schenckii*	Dectin-1 contributes to recognition after GP70 silencing, but the receptor directly responsible for the enhanced NET response has not been established	Conidia and yeast-like cells induce NET formation, with yeast-like cells inducing stronger NET responses	Morphotype-dependent (+)	Gp70-associated masking may limit β-1,3-glucan exposure and neutrophil activation; oxidative-stress adaptation may support fungal persistence, but its direct contribution to NET escape remains unproven	(Galván-Hernández et al., 2023; López-Ramírez et al., 2024; Ruiz-Baca et al., 2019; Félix-Contreras et al., 2020; Saucedo-Campa et al., 2022)
*Fonsecaea pedrosoi*	TLR2/TLR4 mediate conidial recognition, phagocytosis, and ROS production but are not required for hyphal NET-mediated killing	Conidia are controlled mainly through TLR2/TLR4-dependent phagocytosis and ROS production; hyphae induce TLR2/TLR4- and NOX-independent NET release	Morphotype-dependent: hyphae induce NETs, whereas conidia do not in the experimental model examined (+)	DHN-melanin suppresses neutrophil ROS production and NET formation. Zinc-starvation-induced cell-wall remodeling and antioxidant responses may support fungal adaptation to NET-associated nutritional restriction, but their direct contribution to NET evasion has not been established	(Breda et al., 2020; Santos et al., 2024; Zheng et al., 2025; Huang et al., 2024)
*Paracoccidioides brasiliensis*	Specific NET-inducing receptors have not been fully defined	Human neutrophils form NETs against both conidia and yeast cells; NET morphology and integrity vary between fungal isolates	NET-inducing (+), with isolate-dependent NET patterns	A virulent isolate exhibits DNase-like activity that produces looser and degraded NET structures and facilitates fungal escape; the specific protein responsible has not been definitively identified	(Mejía et al., 2015; Zonta et al., 2020)

CR3, complement receptor 3; DHN-melanin, 1,8-dihydroxynaphthalene melanin; GAG, galactosaminogalactan; GXM, glucuronoxylomannan; GXMGal, glucuronoxylomannogalactan; NETs, neutrophil extracellular traps; NF-κB, nuclear factor κB; NOX, NADPH oxidase; PAD4, peptidylarginine deiminase 4; ROS, reactive oxygen species; 3′-NT/NU, 3′-nucleotidase/nuclease. Symbols: ++, strong response; +, detectable response. Qualifiers indicate context-, morphotype-, strain-, or isolate-dependent responses.

## 7 Regulatory networks shaping NET functional outcomes

### 7.1 Local tissue microenvironmental regulation

In a murine model of chronic vulvovaginal candidiasis (VVC), NET formation was associated with enhanced *C. albicans* killing and subsequent fungal clearance, whereas impaired NET formation despite substantial neutrophil recruitment was accompanied by reduced antifungal activity and persistent vaginal fungal burden (Yano and Fidel, 2024). Vaginal heparan sulfate (HS) inhibited NET formation and neutrophil antifungal activity, whereas heparanase treatment restored these responses (Yano and Fidel, 2024). These findings indicate that local microenvironmental factors can impair NET-mediated antifungal defense despite robust neutrophil recruitment, thereby contributing to persistent infection.

In pulmonary aspergillosis, tissue hemorrhage can further alter NET responses through the release of extracellular heme. Experimental evidence showed that extracellular heme synergized with *A. fumigatus* exposure to enhance NET formation and lung injury, whereas disruption of NETs attenuated both tissue injury and fungal burden (Qu et al., 2025). Hemopexin-mediated heme scavenging limited these detrimental responses, supporting a pathological feedback loop between hemorrhage, extracellular heme, and excessive NET formation (Qu et al., 2025).

In an experimental *S. aureus–C. albicans* co-infection model, *S. aureus* can adhere to and surround *C. albicans*, thereby altering its exposure to NET-mediated killing. NETs exhibited stronger antifungal activity against *C. albicans* alone, whereas during co-infection they preferentially targeted the surrounding *S. aureus*, indirectly protecting the enclosed fungal cells (Jing et al., 2024). In the presence of neutrophils, *S. aureus* also promoted *C. albicans* proliferation and hyphal growth, illustrating how interkingdom interactions can modify the outcome of NET-mediated immunity (Jing et al., 2024).

In an experimental *K. pneumoniae–C. albicans* co-infection model, mixed biofilms exhibited greater biomass but induced weaker neutrophil activation and lower NET formation than *K. pneumoniae* biofilms alone (Phuengmaung et al., 2024). Consistently, co-infected mice exhibited reduced inflammatory responses, lower pulmonary NET signals, and less severe lung injury than mice infected with *K. pneumoniae* alone (Phuengmaung et al., 2024). However, whether reduced NET formation directly contributes to the attenuated lung injury remains to be established.

Collectively, these findings indicate that the local tissue microenvironment can shift NET responses toward either insufficient antifungal activity or excessive inflammatory injury, depending on the infectious context.

### 7.2 Metabolic adaptation and maintenance of NET function

The metabolic state of neutrophils is an important determinant of NET formation. Human neutrophils undergo dynamic shifts in nutrient utilization during activation: resting neutrophils rely substantially on intracellular glycogen, whereas activated neutrophils increase glucose uptake and extracellular glucose utilization (Britt et al., 2024). Different effector functions show distinct metabolic requirements, with oxidative burst relying predominantly on extracellular glucose, whereas NET release exhibits greater flexibility in the use of glycogen-derived and extracellular glucose-derived substrates (Britt et al., 2024). Earlier work demonstrated that PMA-induced NET formation is strongly dependent on glucose and glycolysis, with glycolytic inhibition markedly suppressing NET release (Rodríguez-Espinosa et al., 2015). Together, these findings establish metabolic substrate availability as an important regulator of NET formation, although how such metabolic flexibility influences NET-mediated antifungal defense remains incompletely understood.

In metabolic disorders, abnormal nutrient signaling may disrupt NET functional balance. Hyperglycemic conditions associated with type 2 diabetes induce metabolic reprogramming in neutrophils, promote constitutive NET formation, and reduce subsequent NET responses to LPS stimulation (Joshi et al., 2020). Neutrophils under diabetic conditions also exhibit enhanced glycolysis, pentose phosphate pathway activity, and fatty acid oxidation, together with histone acetylation that primes them for NET formation through a trained-immunity mechanism (Shrestha et al., 2024). These findings indicate that metabolic dysregulation can simultaneously promote basal NET activation and alter subsequent neutrophil responsiveness. However, whether these diabetes-associated metabolic and epigenetic changes directly modify NET-mediated antifungal immunity remains to be established.

### 7.3 Molecular checkpoints regulating NET functional states

The myeloid inhibitory C-type lectin-like receptor (MICL) is an inhibitory receptor expressed on myeloid cells that regulates neutrophil activation. A recent study identified MICL as a receptor that directly recognizes DNA within NETs and limits further NET formation through suppression of the ROS–PAD4 pathway, thereby establishing a negative-feedback mechanism that restrains excessive neutrophil activation (Malamud et al., 2024).

The functional consequences of this regulatory pathway are context dependent. In response to *A. fumigatus*, MICL-deficient neutrophils exhibited enhanced NET formation, while MICL-deficient mice showed improved survival and reduced fungal burden following systemic infection (Malamud et al., 2024). Importantly, pharmacological inhibition of PAD4 reversed this enhanced resistance in MICL-deficient mice, supporting a contribution of increased NET formation to improved fungal control (Malamud et al., 2024). These findings illustrate that a regulatory pathway that limits excessive NET formation may protect against inflammatory pathology in some settings but can also constrain protective antifungal immunity.

Thus, MICL may represent a molecular checkpoint linking NET homeostasis to context-dependent immune outcomes. However, whether MICL can be therapeutically modulated to enhance antifungal defense without promoting excessive inflammation remains unknown.

In contrast to MICL-mediated negative feedback regulation, the modification status of certain host-derived molecules may promote the transition of NET responses toward a pro-inflammatory state.

Host-derived damage-associated molecular patterns (DAMPs) not only influence the extent of NET formation but may also reshape the functional states of NET responses. Extracellular vimentin (eVim) and its citrullinated form (CitVim) may also differentially regulate neutrophil effector states. In human neutrophils, both forms activated TLR4, whereas CitVim induced stronger ROS production and NET formation through a predominantly TLR4- and ROS-dependent mechanism (Suprewicz et al., 2025). In contrast, eVim more effectively enhanced phagocytosis and intracellular killing of *C. albicans* than CitVim (Suprewicz et al., 2025). These findings suggest that post-translational modification of extracellular proteins may shift neutrophil responses between distinct effector programs; however, whether CitVim-induced NET formation directly alters antifungal outcomes remains unknown.

### 7.4 Cell-intrinsic programmatic regulation

Recent evidence has identified XKR8, a plasma membrane phospholipid scramblase, as a cell-intrinsic regulator of NET formation. Following NET-inducing stimulation, caspase-3-mediated cleavage activates XKR8, triggering phospholipid scrambling, altered membrane tension, and subsequent Ca²^+^ signaling through mechanosensitive channels (Liu et al., 2026). XKR8 deficiency markedly impaired NET formation in response to multiple stimuli, including *C. albicans*, and compromised fungal control in a murine pulmonary candidiasis model (Liu et al., 2026). These findings identify the caspase-3–XKR8–membrane scrambling axis as a regulatory mechanism linking intracellular NET signaling to protective antifungal immunity.

Although lytic NETosis and apoptosis have traditionally been regarded as distinct cell-death programs, recent evidence suggests that apoptotic signaling can also engage NET-forming mechanisms.

GSDME has been implicated in the coupling of apoptotic signaling to NET formation. GSDME-dependent Ca²^+^ mobilization and membrane permeabilization promote PAD4 activation, histone citrullination, and nuclear DNA externalization in apoptotic neutrophils (Zhu et al., 2023). However, whether this GSDME-dependent program contributes to NET formation or antifungal defense during fungal infection remains unknown.

Collectively, NET outcomes during fungal infections are shaped by fungal characteristics, host metabolic and immune states, local microenvironmental factors, and cell-intrinsic regulatory pathways. Understanding these interacting determinants may help explain discrepancies among studies and inform the development of context-specific NET-targeted strategies.

## 8 Clinical translation of NET biology in fungal infections

Conventional antifungal agents remain indispensable for the management of invasive fungal infections, but their effectiveness can be limited by antifungal resistance, drug toxicity, and the restricted spectrum of available therapies (Denning, 2003; Lu et al., 2025). These limitations have increased interest in complementary host-directed approaches. Given the dual protective and pathological roles of NETs in fungal infections, NET-associated immune responses have emerged as potential targets for therapeutic modulation.

### 8.1 NET-associated biomarkers

NETs have been directly detected in corneal scrapings from patients with fungal keratitis. In a small clinical study of 14 patients, greater NET formation was associated with better treatment responses and shorter disease courses, suggesting a relationship between local NET responses and clinical outcome (Jin et al., 2016). Consistent with this association, dexamethasone treatment in a murine *C. albicans* keratitis model reduced neutrophil infiltration and NET formation while increasing fungal burden and disease severity (Fan et al., 2020). However, these findings establish associations between NET abundance and disease outcome rather than validating NETs as prognostic biomarkers. The small clinical sample size, lack of standardized thresholds, and absence of established diagnostic performance currently limit their clinical application.

More quantitative diagnostic evidence has been reported for synovial fluid NETs (SF-NETs) in periprosthetic joint infection (PJI). SF-NET levels showed high diagnostic performance for PJI overall, with an area under the receiver operating characteristic curve of 0.971, a sensitivity of 93.48%, and a specificity of 96.43% (Cai et al., 2023). Subgroup analyses further suggested potential diagnostic utility in fungal PJI (Cai et al., 2023). However, these findings were derived from a single-center cohort with a relatively small sample size, and the unusually high proportion of fungal PJI cases may have influenced the overall diagnostic estimates. Independent multicenter validation is therefore required before SF-NETs can be considered a clinically established biomarker.

In clinical studies, NET formation is generally assessed using surrogate markers rather than by directly measuring the dynamic process of NETosis. Commonly used markers include cfDNA, dsDNA, nucleosomes, CitH3/CitH4, and MPO-DNA or NE-DNA complexes, although their specificity for NET formation varies substantially (Stoimenou et al., 2022). For fluid samples, molecular assays are commonly used, whereas immunofluorescence demonstrating colocalization of extracellular DNA with NET-associated proteins such as CitH3, MPO, or NE provides stronger structural evidence of NET formation (Stoimenou et al., 2022). Notably, cfDNA can also originate from apoptotic or necrotic cells, while MPO and NE may be released through neutrophil degranulation independently of NET formation; even MPO-DNA assays may show limited specificity *in vivo (*Stoimenou et al., 2022). Consequently, standardized criteria for NET quantification and interpretation remain lacking (Stoimenou et al., 2022).

In addition to NET-associated parameters, currently available non-culture biomarkers for the auxiliary diagnosis of invasive fungal infections mainly include β-D-glucan (BDG), galactomannan (GM), mannan antigen/anti-mannan antibodies, and fungal nucleic acid detection assays (Pappas et al., 2016; Patterson et al., 2016; Cornely et al., 2025). These approaches primarily provide pathogen-derived information, including fungal cell wall polysaccharides, antigens, or nucleic acids, and have important clinical value in the auxiliary diagnosis of invasive candidiasis and invasive aspergillosis (Pappas et al., 2016; Patterson et al., 2016; Cornely et al., 2025). However, their applicability and diagnostic performance vary considerably and can be influenced by multiple factors, including host characteristics, specimen type, antifungal therapy, and assay platforms (Patterson et al., 2016; Cornely et al., 2025). For example, GM testing is mainly applied for the diagnosis of invasive aspergillosis in selected high-risk populations; BDG provides broad-spectrum fungal detection but lacks species-level identification; mannan/anti-mannan assays are mainly used for invasive candidiasis; and the clinical performance of fungal PCR assays depends on detection platform, specimen type, and methodological standardization (Pappas et al., 2016; Patterson et al., 2016; Cornely et al., 2025).

In contrast to pathogen-derived fungal biomarkers, NET-associated parameters primarily reflect host immune activation and NET formation status (Patterson et al., 2016; Stoimenou et al., 2022). However, NET formation is not specific to fungal infections and can also occur in other infectious and sterile inflammatory conditions (Stoimenou et al., 2022). Therefore, NET-associated parameters alone are unlikely to provide sufficient specificity for fungal pathogen identification. Currently, NET-related biomarkers may be better considered as potential adjunctive host-response biomarkers rather than replacements for established fungal diagnostic assays such as BDG and GM. Future studies should determine whether combining pathogen-derived biomarkers with NET-associated parameters can improve diagnostic accuracy or provide additional prognostic value. Such approaches will require validation in large, multicenter prospective cohorts using standardized NET assays and predefined diagnostic thresholds.

### 8.2 NET-targeted therapeutic strategies

Given the dual roles of NETs in fungal infections, NET-targeted therapies should aim to achieve functional balance rather than simply enhance or suppress NET formation. Current strategies can be broadly categorized into enhancing protective NET-associated antifungal immunity, limiting pathological NET-driven inflammation, and developing NET-inspired therapeutic approaches.

GM-CSF is a clinically investigated host-directed immunomodulator with potential relevance to NET-associated antifungal immunity. In a prospective multicenter randomized trial involving recipients of allogeneic hematopoietic stem-cell transplantation, GM-CSF administration was associated with improved responses to antifungal therapy and reduced mortality from invasive fungal disease (Wan et al., 2015). Mechanistically, GM-CSF enhances neutrophil antifungal functions, including oxidative responses, and GM-CSF signaling contributes to pulmonary defense against *A. fumigatus (*Kasahara et al., 2016; Dong et al., 2025; Mills et al., 2025).

Recent evidence further indicates that epithelial-derived GM-CSF promotes neutrophil recruitment and antifungal activity during pulmonary aspergillosis (Mills et al., 2025).

However, GM-CSF should not currently be considered a direct NET-targeted therapy. Although enhanced neutrophil activation and ROS production may influence NET formation, direct evidence that GM-CSF therapeutically modulates NET formation during fungal infection remains limited. Thus, its relevance to NET biology is better viewed as indirect host-directed immunomodulation rather than selective targeting of NETosis.

In immunosuppressed models of *C. auris* infection, adjunctive GM-CSF enhanced neutrophil recruitment and activation, ROS production, and NET-associated antifungal responses, accompanied by reduced fungal burdens in multiple organs (Mattos et al., 2025). Although these findings suggest that GM-CSF can enhance NET-associated antifungal responses in *C. auris* infection, direct evidence establishing NET regulation as a major mechanism underlying its therapeutic effects remains limited. Moreover, clinical evidence supporting adjunctive GM-CSF therapy for invasive fungal diseases is largely derived from selected patient populations and small observational studies (Chen et al., 2022). Further studies are needed to determine how GM-CSF influences NET formation and function across different fungal pathogens and host immune states.

In addition, targeting fungal immune evasion mechanisms may provide new opportunities to enhance host antifungal immunity. The melanin biosynthesis inhibitor tricyclazole (TCZ) suppressed DHN-melanin production in *Fonsecaea pedrosoi* and enhanced neutrophil ROS production and NET formation *in vitro (*Zheng et al., 2025). In a murine model of chromoblastomycosis, TCZ treatment also reduced local inflammation and fungal burden (Zheng et al., 2025). These findings suggest that targeting fungal virulence mechanisms that suppress neutrophil function may help restore protective NET-associated antifungal responses.

In *Candida albicans* biofilms, pharmacological inhibition of HIF-1α with LW6 or PX478 partially restored neutrophil ROS production and NET formation, suggesting that modulation of HIF-1α signaling may help reverse biofilm-associated impairment of NET-mediated antifungal defense (Juszczak et al., 2025). However, the therapeutic relevance of this approach in clinical fungal infections requires further validation.

Beyond direct modulation of endogenous NETs, NET-inspired biomaterials may provide conceptual frameworks for future therapeutic development. For example, NETosis-inspired framework nucleic acid traps (FNATs) have been engineered to form stimulus-responsive, web-like DNA networks on target cell surfaces, enabling extracellular recognition, cellular trapping, and localized effector delivery (Gong et al., 2024). However, this platform has been investigated primarily in tumor-related models, and its applicability to fungal pathogens or antifungal therapy has not yet been established.

The dual protective and pathological roles of NETs create a particular challenge for NET-directed therapy in fungal infections. Experimental disruption of NETs with DNase I has reduced NET-dependent antifungal activity in specific *C. albicans* models, particularly against hyphal forms (Kenno et al., 2016), while NET degradation also reverses NET-mediated restriction of *A. fumigatus* germ-tube growth (McCormick et al., 2010). These findings suggest that indiscriminate NET degradation could potentially interfere with protective extracellular antifungal responses. In this context, nanotechnology may be particularly useful because it can provide greater spatial and cellular control over the delivery of NET-modulating agents, rather than relying on broad NET suppression. Lee et al. developed a sequential nanoparticle strategy combining local pulmonary delivery of DNase I-loaded polydopamine nanoparticles for NET clearance with neutrophil-targeted delivery of sivelestat-loaded PLGA nanoparticles to suppress neutrophil elastase activity and excessive neutrophil activation (Lee et al., 2025). This strategy illustrates how local and cell-directed delivery can be incorporated into NET-targeted intervention. Although this approach was evaluated in non-fungal pulmonary inflammatory models, its delivery principle may be relevant to fungal infections, where NETs can contribute to both antifungal defense and inflammatory tissue injury. Spatially or cell-selective delivery of NET-modulating agents could therefore provide a strategy to preferentially control pathological NET accumulation while minimizing systemic interference with protective neutrophil functions. However, whether such an approach can preserve NET-mediated antifungal activity while limiting pathological NET responses has not yet been directly demonstrated in fungal infection models and requires further investigation.

The differential involvement of PI3K isoforms in *H. capsulatum*-induced NET formation further suggests that isoform-specific modulation of PI3K signaling may provide a potential strategy for more selective regulation of NET responses (Thompson-Souza et al., 2026). However, whether such an approach can modulate pathological NET formation while preserving antifungal activity remains to be determined.

Gene-editing studies further indicate that NET formation is not the sole determinant of antifungal defense. PTP1B-knockout iPSC-derived neutrophils showed reduced ROS production and NET formation but enhanced migration, phagocytosis, and control of *A. fumigatus (*Giese et al., 2024). These findings support selective modulation of neutrophil functions rather than global enhancement of NET formation, as antifungal defense relies on multiple complementary neutrophil effector mechanisms (Gazendam et al., 2016; Giese et al., 2024).

Although XKR8-mediated phospholipid scrambling represents a potential molecular checkpoint for NET modulation, XKR8 deficiency impairs NET formation and compromises fungal control in experimental *C. albicans* infection (Liu et al., 2026). Therefore, therapeutic inhibition of XKR8 could also weaken protective antifungal immunity, and whether this pathway can be selectively targeted to limit pathological NET responses without compromising host defense remains to be determined.

However, the clinical application of NET-targeted therapies should account for the underlying immune status of patients. Prolonged or profound neutropenia is an established risk factor for invasive fungal infections, particularly invasive aspergillosis in hematological populations (Freifeld et al., 2011). However, invasive pulmonary aspergillosis can also occur in the absence of prolonged neutropenia, emphasizing the heterogeneity of host susceptibility (Abers et al., 2016). Because NET formation requires functional neutrophils, profound neutropenia would be expected to limit the capacity to generate protective NET responses (Papayannopoulos, 2018). In this setting, broad suppression of NET formation may provide limited benefit and could further compromise residual neutrophil-mediated antifungal defense. Accordingly, NET-targeted interventions should be adapted to the immune status of individual patients rather than applied uniformly (Espiritu and O'Sullivan, 2025).

Future NET-targeted therapies should focus on context-specific modulation rather than universal promotion or inhibition of NET formation. Selective inhibition of pathological NET responses may be beneficial when excessive NET activation contributes to inflammatory tissue injury, whereas preservation or restoration of neutrophil-mediated antifungal defense should take priority in patients with impaired neutrophil function. Because therapeutic responses may also vary with the degree of immunosuppression, tissue barrier integrity, and pathogen-specific characteristics, patient stratification will be essential for the development of clinically applicable NET-targeted strategies.

## 9 Discussion: current challenges and future perspectives

Despite substantial experimental progress, the clinical translation of NET-targeted strategies in fungal infections remains limited. Most mechanistic evidence is derived from *in vitro* systems and animal models that do not fully recapitulate the complexity of human fungal diseases, while candidate NET-directed interventions may also be constrained by off-target effects, delivery limitations, and potential interference with antimicrobial host defense (Papayannopoulos, 2018; Espiritu and O'Sullivan, 2025). These limitations highlight the need for clinically relevant models and rigorous safety evaluation before NET-modulating strategies can be translated into practice.

More fundamentally, clear criteria for distinguishing protective from pathological NET responses remain lacking. Rather than representing two discrete states, these functional outcomes likely exist along a dynamic continuum shaped by fungal species and morphology, the magnitude and duration of NET formation, fungal immune-evasion mechanisms, the local tissue microenvironment, and host immune status. Within this framework, fungal recognition and downstream signaling initiate NET formation, while fungi may evade, suppress, or resist NET-mediated immunity, and host- and tissue-derived factors further modify the magnitude and functional consequences of the response. The interaction of these factors ultimately determines whether NETs primarily contribute to fungal trapping, growth restriction, and clearance or instead promote persistent inflammation and tissue injury. Importantly, specific molecular markers capable of reliably distinguishing protective from pathological NET responses have not yet been identified.

**Figure 2:**
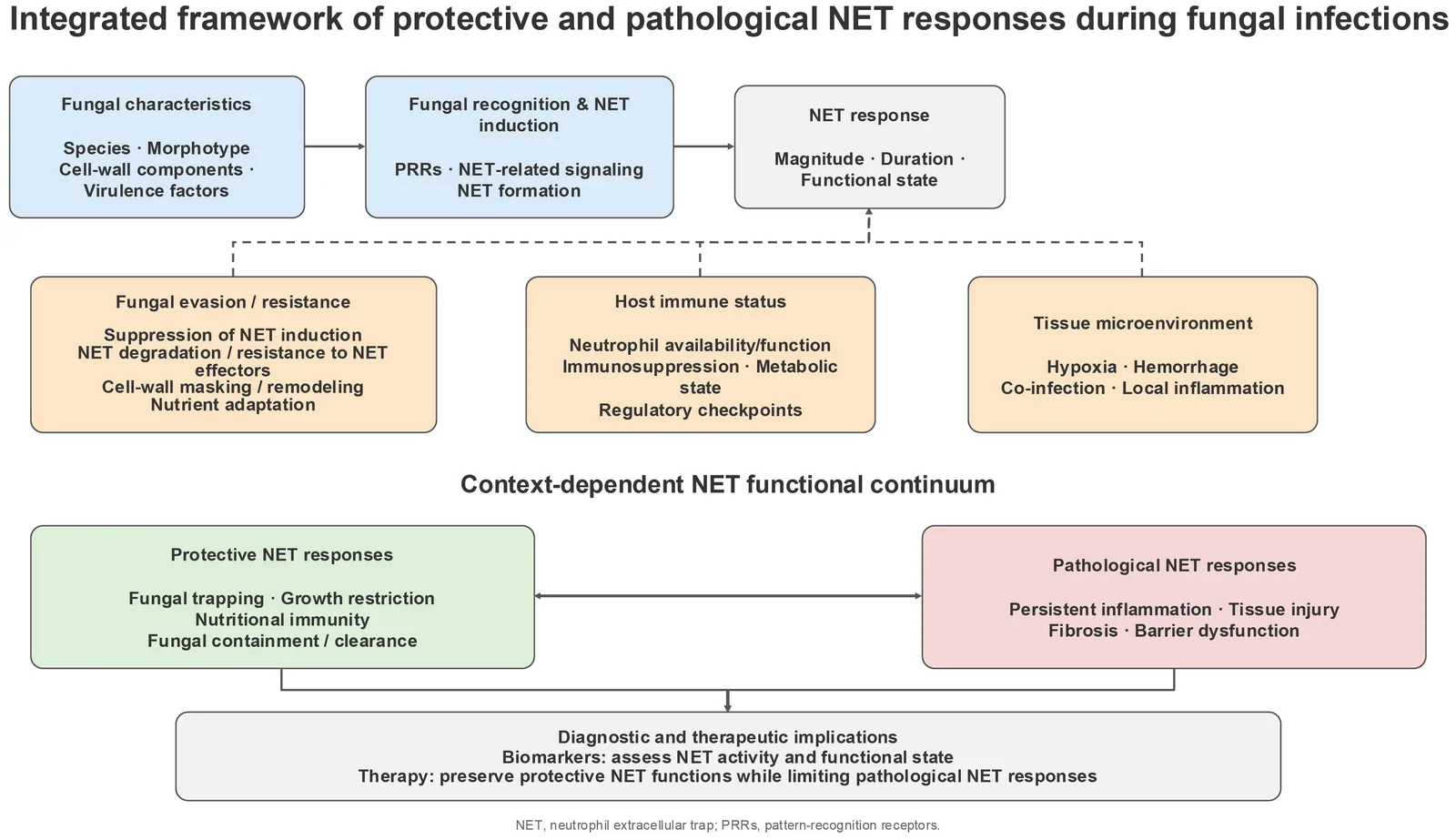
Integrated framework of protective and pathological NET responses during fungal infections.

Future research should therefore focus on defining the molecular and environmental determinants that shift NET responses from protective fungal containment toward pathological inflammation. Intravital imaging and single-cell approaches may help resolve the spatial and temporal regulation of NET responses across different fungal pathogens, tissues, and host immune states. Integrating these mechanistic insights with the identification of reliable NET-associated biomarkers and regulatory checkpoints will be essential for developing diagnostic and therapeutic strategies that can distinguish pathological NET activity from protective antifungal immunity and selectively target the former while preserving the latter (**Figure 2**).
